# Antitumor effect of free rhodium (II) citrate and rhodium (II) citrate-loaded maghemite nanoparticles on mice bearing breast cancer: a systemic toxicity assay

**DOI:** 10.1007/s13277-014-2966-x

**Published:** 2014-12-21

**Authors:** Raphael Cândido Apolinário Peixoto, Ana Luisa Miranda-Vilela, José de Souza Filho, Marcella Lemos’ Brettas Carneiro, Ricardo G. S. Oliveira, Matheus Oliveira da Silva, Aparecido R. de Souza, Sônia Nair Báo

**Affiliations:** 1 Institute of Biological Sciences, Department of Cell Biology, University of Brasília (UnB), Brasilia, 70919-970 Brazil; 2 Institute of Biological Sciences, Department of Genetics and Morphology, University of Brasilia (UnB), Brasília, DF 70910-900 Brazil; 3 Faculty of Medicine, Faciplac, Campus Gama, Gama, DF Brazil; 4 Institute of Chemistry, Federal University of Goiás, Campus Samambaia, Goiânia, Goiás 74001-970 Brazil

**Keywords:** Rhodium (II) citrate, Maghemite nanoparticles, 4T1 breast cancer, Balb/c mice, Toxicological analysis

## Abstract

Breast cancer is one of the most prevalent cancer types among women. The use of magnetic fluids for specific delivery of drugs represents an attractive platform for chemotherapy. In our previous studies, it was demonstrated that maghemite nanoparticles coated with rhodium (II) citrate (Magh-Rh_2_Cit) induced in vitro cytotoxicity and in vivo antitumor activity, followed by intratumoral administration in breast carcinoma cells. In this study, our aim was to follow intravenous treatment to evaluate the systemic antitumor activity and toxicity induced by these formulations in Balb/c mice bearing orthotopic 4T1 breast carcinoma. Female Balb/c mice were evaluated with regard to toxicity of intravenous treatments through analyses of hemogram, serum levels of alanine aminotransferase, iron, and creatinine and liver, kidney, and lung histology. The antitumor activity of rhodium (II) citrate (Rh_2_Cit), Magh-Rh_2_Cit, and maghemite nanoparticles coated with citrate (Magh-Cit), used as control, was evaluated by tumor volume reduction, histology, and morphometric analysis. Magh-Rh_2_Cit and Magh-Cit promoted a significant decrease in tumor area, and no experimental groups presented hematotoxic effects or increased levels of serum ALT and creatinine. This observation was corroborated by the histopathological examination of the liver and kidney of mice. Furthermore, the presence of nanoparticles was verified in lung tissue with no morphological changes, supporting the idea that our nanoformulations did not induce toxicity effects. No studies about the systemic action of rhodium (II) citrate-loaded maghemite nanoparticles have been carried out, making this report a suitable starting point for exploring the therapeutic potential of these compounds in treating breast cancer.

## Introduction

Breast cancer is the most common type of cancer among women (excluding non-melanoma skin cancer) in both developed and developing countries [1]. Conventional treatments include surgery, radiotherapy, chemotherapy, and hormone therapy, and their effectiveness depends on the progress and type of the tumor [2, 3]. However, breast cancer remains one of the world’s most devastating diseases, with more than 458,000 deaths and 1.38 million new cases each year [4, 5]. Chemotherapy, which is a common therapeutic approach, is used in high doses due to its lack of specificity to tumor cells, with consequent high systemic toxicity [6].

Therefore, the development of therapeutic strategies to selectively deliver drugs to the tumor, with no toxic effects on healthy tissues, represents an attractive area for breast cancer treatment. Among them, nanotechnology has been indicated as a new approach for cancer treatment over the past decade. It is starting to be used to develop imaging agents and nanosensors that detect biological signs of cancer and nanoparticles (NPs) that target cancerous cells with drugs [8]. The latter direction represents progress in relation to traditional therapies, since it would reduce systemic toxicity, thanks to the higher efficiency of drug delivery in chemotherapy. Indeed, superparamagnetic nanoparticles (SPIOs) mediating drug delivery, for instance, are used to target and accumulate drug molecules in the tumor, increasing the effectiveness of chemotherapy [7–9].

The small size of SPIOs has an influence on the passive transport of drugs through the tumor vessels and determines the speed at which they are removed by the reticuloendothelial system (RES). Firstly, SPIOs may have good physiological tolerance if they are functionalized with hydrophilic agents, preventing the opsonization by plasma protein [10–12]. Second, tumors larger than 2 mm^3^ have an increased demand for blood, resulting in the process of angiogenesis and leaky vasculature with 100–800-nm pores, larger than those found in healthy blood vessels [8]. This defective architecture impairs lymphatic drainage near the tumor, increasing the enhanced permeability and retention (EPR) effect of SPIOs in the tumor [13, 14]. Finally, SPIO-mediated drug delivery has the potential to enhance drug bioavailability, improve the time release of drug molecules, and enable precision drug targeting. It represents progress in relation to traditional therapies by reducing the systemic toxicity [7–9, 12, 15–17].

A variety of SPIOs coated with chemotherapy agents, including some inorganic compounds such as cisplatin and carboplatin, have been studied [18, 19]. Free rhodium (II) citrate (Rh_2_Cit) is also an inorganic and antitumor compound [20], which has been successfully stabilized by maghemite NPs [21]. Moreover, Rh_2_Cit presents hydrophilic groups, which improve its internalization by tumor cells and reduce the uptake by the RES [22].

Our research group has demonstrated that Rh_2_Cit and rhodium (II) citrate-loaded maghemite nanoparticles (Magh-Rh_2_Cit) induced significant cytotoxic effects on 4T1 and MCF-7 breast cancer cell lines. Moreover, these formulations reduced orthotopic 4T1 metastatic mammary carcinoma by intratumoral administration in Balb/c mice [23, 24]. Local injection therapy as intratumoral drug administration is commonly used for studying drug efficacy against non-metastatic tumors [25]. It results in less non-specific cytotoxic activity than with systemic treatments, which can induce adverse effects [26]. However, it is important to investigate the effect of the treatments by intravenous administration (i.v.), since this is one of the routes more frequently used in clinical treatment against breast metastatic cancer [6, 27]. Furthermore, no studies on the systemic action of Rh_2_Cit and Magh-Rh_2_Cit have been carried out, making this report a suitable starting point for exploring the therapeutic potential of these compounds in treating breast cancer.

Thus, we aimed to investigate the effect of systemic treatment with Rh_2_Cit and Magh-Rh_2_Cit on Balb/c mice bearing 4T1 mammary carcinoma. Maghemite nanoparticles coated with citrate (Magh-Cit) were used as control. The 4T1 cell line is suitable as an experimental animal model since it develops metastasis spontaneously from primary tumor, similar to human breast cancer, and also enables a rapid orthotopic tumor growth in Balb/c mice [28]. Moreover, the orthotopic model is appropriate for understanding the interaction of the drug with the tumor in its original microenvironment, being useful to recognize how cancer cells respond to the immune system, blood, hormones, and growth factors [29, 30]. For this purpose, the use of NPs (Magh-Rh_2_Cit and Magh-Cit) could improve drug delivery to the tumor to make the most effective cancer treatment [8, 9, 11, 22].

## Materials and methods

### Animals and procedures

A total number of 40 Balb/c female mice (12 weeks old) were purchased from the Multidisciplinary Center for Biological Investigation in Laboratory Animal Science (Cemib) of the State University of Campinas (Unicamp, SP/Brazil). All mice were maintained in plastic cages under standard conditions of 12-h dark/light cycle. The animals, weighing at the beginning of the experiment 21.83 ± 1.52 g, were fed with standard diet and water ad libitum.

Mice were evaluated with regard to the treatments’ toxicity through analyses of blood, liver, kidney, and lung histology. In addition, the antitumor activity of rhodium (II) citrate and maghemite nanoparticles coated with rhodium (II) citrate was verified by tumor volume reduction, morphometry, and histology. All experiments described were approved by the Animal Research Ethics Committee of the University of Brasilia–Institute of Biologic Sciences, Brazil (UnBDOC no. 109434/2008).

### Preparation of the rhodium (II) citrate and rhodium (II) citrate-loaded maghemite nanoparticles

All formulations (Rh_2_Cit, Magh-Rh_2_Cit and Magh-Cit) were prepared and characterized as previously described [23, 24]. Magh-Cit was used as control of NPs without rhodium. Briefly, Rh_2_Cit was synthesized by exchanging trifluoroacetate ligands from the precursor rhodium (II) trifluoroacetate with citrate ligands. The compound was obtained as a green aqueous solution with a standardized concentration of 0.054 mol/L. Maghemite nanoparticles were synthesized by alkaline co-precipitation of Fe^2+^ and Fe^3+^ ions [31]. The particles obtained in the magnetite (Fe_3_O_4_) phase were oxidized to maghemite (γ-Fe_2_O_3_) by bubbling of oxygen gas and were subsequently purified by dialysis with deionized water for several days ([Fe] = 0.37 M). The Magh-Rh_2_Cit was prepared using 5 mL of the colloidal dispersion with 1 mL of Rh_2_Cit and stirred for 24 h.

### Orthotopic tumor cell implantation and treatment

The 4T1 breast carcinoma cells were thawed and cultivated in flasks with Dulbecco’s modified Eagle’s medium (DMEM) (Sigma, St. Louis, MO) supplemented with 1 % penicillin and 10 % fetal bovine serum (FBS) at 37 °C in a humidified atmosphere with 5 % CO_2_. One week later, Balb/c mice were anesthetized with ketamine (80 mg/kg) and xylazine (10 mg/kg) by intraperitoneal route. Then, 2 × 10^4^ 4T1 cells (in suspension in 50 μL serum-free DMEM) were injected (1-mL-gauge needle) in their left mammary gland, which is the natural primary microenvironment of breast tumor occurrence.

Seven days after implantation of 4T1 cells, mice bearing tumor were distributed into four experimental groups (*n* = 8/group), and each group was treated with 50 μL of (1) Rh_2_Cit, (2) Magh-Rh_2_Cit, (3) Magh-Cit, or (4) water (solution solvent, control group). Treatments were carried out every 4 days by i.v. injection in the animal’s tail vein, totalizing three applications of 0.5 mg/kg rhodium (II) citrate for each one (total dose of Rh_2_Cit was 1.5 mg/kg). Mice treated with Magh-Cit received the same iron concentration and nanoparticle amount found in Magh-Rh_2_Cit (14 mg/kg and 2.4 × 10^15^ particles). It were also included animals without tumor (healthy, *n* = 8) for comparison with other groups exhibiting tumor.

During the experiments, deaths were registered for all mice bearing tumor, leaving at least five animals per group. Thus, three or five mice were used in each group for the following analysis. On the 19th day after tumor implantation, 4 days after the last treatment, mice were anesthetized as previously described, and blood samples (1 mL/animal) were collected by cardiac puncture to carry out hemogram and biochemical dosages of alanine aminotransferase (ALT), creatinine and serum iron. The animals were then immediately euthanized by cervical dislocation, according to the American Veterinary Medical Association (AVMA) guidelines for euthanasia [32]. Then tumor, liver, kidney, and lung were collected to perform antitumor and systemic toxicity analysis as described below.

### Tumor regression

Tumors were surgically removed, their width and length were measured by a digital pachymeter (Stainless, Hardened), and the tumor volume was calculated according to the formula length × width^2^ × 0.52 [33]. The tumor regression in experimental groups was evaluated by the tumor’s volume, histopathology, and morphometry.

Tumors were fixed in 4 % paraformaldehyde diluted in phosphate-buffered saline (PBS) for 4 hours at room temperature, transferred to 70 % ethanol, included in paraffin using an automatic tissue processor (OMA® DM-40, São Paulo, Brazil), cut to 5 μm of thickness in a Leica RM2235 manual microtome (Leica Microsystems, Nussloch, Germany) and stained with hematoxylin-eosin (HE) for histological analyses (light microscopy), in order to verify cell proliferation pattern, pleomorphism, degree of cell differentiation, and cell death. Morphometric analysis was also carried out to verify the percentage of coagulation necrosis for each treatment. Necrosis areas were measured as adapted from our previous reports [8, 37], with a total of three sections (20 μm far) for each tumor collected from five mice per group.

### Systemic toxicity analysis in mice bearing 4T1 breast cancer after intravenous injection of compounds

In order to evaluate potential systemic toxicity induced by the treatments, clinical, histology and blood analyses were performed. Treated mice were continuously monitored for toxicity indexes such as weight loss, diarrhea, skin ulcers, and deaths; the time of animal death was recorded throughout the experimental period.

Hemogram was processed in a multiple automated hematology analyzer (XZ 2100 Sysmex equipment), and serum biochemical analyses were run on the automated chemistry analyzer ADVIA 2400 (Siemens), using the appropriate Advia chemistry reagents, protocols, and controls.

Histopathology analysis of the liver, kidneys, and lungs was also performed in order to verify possible toxic effects induced by treatments. For this, tissues were fixed in Davidson’s solution for 24 h at room temperature, transferred to 70 % ethanol, and processed for histology as described above for tumors.

Possible histopathological alterations in the liver were verified and quantified though morphometry, using the histopathological alteration index (HAI) [34]. They were classified as progressive stages for the deterioration of organ functions: I (do not compromise the functioning of the organ), II (severe, affecting normal body functions), and III (very severe and irreversible). A value of HAI was calculated for each group of animals using the formula HAI = (1 × ΣI) + (10 × ΣII) + (100 × ΣIII). Since I, II, and III correspond to the number of stages of change, the mean HAI was divided into five categories: 0–10 = normal tissue; 11–20 = mild to moderate damage to the tissue; 21–50 = moderate to severe damage to the tissue; 51–100 = severe damage to the tissue; and greater than 100 = irreparable damage to the tissue. A morphometric model [35, 36] was also applied to obtain the volume, area, and diameter parameters of hepatocytes. Images of liver histopathological sections of three animals in each group were made with ×40 objective lens, resulting in nine images per group. Measures of ten hepatocytes for each image were performed using Image Pro-Plus 6.0 software (Media Cybernetics, Silver Spring, USA/Microsoft® Windows 32-bit Systems Window® XP Vista). The volumetric measurements were estimated, considering the hepatocytes as a sphere with a volume *V* = 4/3π*R* ^3^ where the calculation of the radius (*R*) was given by the formula *A* = π*R* ^2^ [37].

### Statistical analysis

Statistical analysis was carried out using SPSS (Statistical Package for the Social Sciences) version 15.0. Data were expressed as mean ± SEM (standard error of mean) and values of *p* < 0.05 were considered statistically significant. The continuous variables were tested for normal distribution with the Shapiro-Wilk test. Differences among the analyzed groups were investigated through ANOVA when the data were normally distributed or through Kruskal-Wallis test in order to identify statistical difference when data were not normally distributed. For significant ANOVA and Kruskal-Wallis results, Bonferroni post-test and Mann-Whitney *U* test were respectively used to carry out 2-by-2 comparisons between the treatments. For significant Kruskal-Wallis results, Mann-Whitney *U* test was performed to verify differences between the treatments (2-by-2 comparisons).

For the morphometric analysis of the liver, differences among groups were checked by ANOVA followed by the Tukey test, using the PDF Word Count & Frequency Statistics Software 7.0. Values of *p* < 0.05 were considered statistically significant.

## Results and discussion

### Tumor dimensions and morphometry

**Fig. 1:**
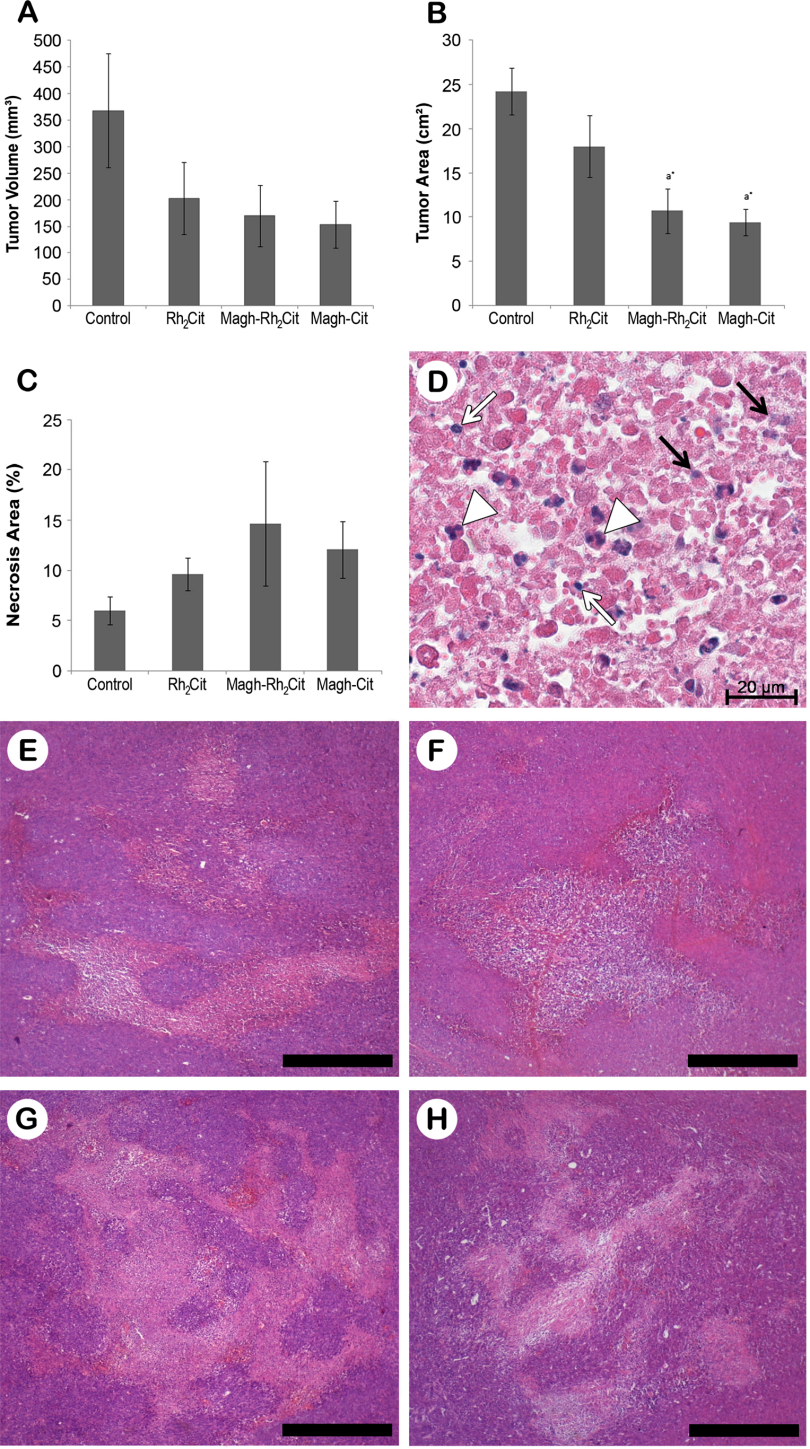
Dimensions, morphometry, and histology of 4T1 tumor tissue sections of the non-treated and treated groups. **a** Tumor volume. **b** Tumor area (morphometry). **c** Percentage of necrosis area (morphometry). **d** Description of coagulation necrosis. **e**–**h** Histopathological aspects by H&E staining, showing a central area of necrotic tissue surrounded by viable tumor cells in the peripheral areas of tumors without treatment (control group, **e**) and those of the treatment groups Rh_2_Cit (**f**), Magh-Rh_2_Cit (**g**), and Magh-Cit (**h**). Pyknotic nuclei (*white arrow*). Karyolysis (*black arrow*). Karyorrhexis (*arrow head*). *Bars* = 0.5 mm

We verified that the tumor growth inhibition in the treatments with Rh_2_Cit, Magh-Rh_2_Cit, and Magh-Cit was about 45, 54, and 58 %, respectively (Fig. 1a). Although these rates were statistically non-significant compared to the control group, the treatments with Magh-Rh_2_Cit and Magh-Cit also promoted a significant decrease in tumor area (Fig. 1b). Thus, Magh-Rh_2_Cit and Magh-Cit could be considered more efficient in retaining tumor aggressiveness and inducing its regression than Rh_2_Cit. This may be related to the hydrophilicity of rhodium (II) citrate [20], which increases its dispersion by the body, especially when the treatments are performed intravenously.

In our previous study, under the same conditions of tumor establishment, mice that were intratumorally treated with 2.0 mg/kg (seven injections of 0.28 mg/kg each dose) of Rh_2_Cit free or associated to NPs (Magh-Rh_2_Cit) presented a mean reduction in the tumor volume of 74 and 52 %, respectively [24]. Thus, in a local application, free rhodium (II) citrate has a higher antitumor effect than rhodium (II) citrate associated with NPs. This corroborates the suggestion that the systemic application of Rh_2_Cit resulted in an increase of its dispersion by the body, probably because of its hydrophilicity [20], and this could have caused a minor tumor reduction when compared to Magh-Rh_2_Cit or Magh-Cit.

Additionally, it is possible that the combination of NPs with Rh_2_Cit may have improved the passive targeting of nanoparticles (Magh-Rh_2_Cit) to the tumor due to the EPR effect in abnormal tissue, which is characterized by a leaky vasculature in combination with poor lymphatic drainage around the tumor [38]. Tumor microvasculature typically contains pores ranging from 100 to 1000 nm in diameter, while in most healthy tissue, there are tight intercellular junctions of less than 10 nm [17]. Therefore, the EPR effect may have caused the accumulation of NPs next to tumor tissues since the hydrodynamic size of the NPs used in the experiments was 60 nm [21]. This size is greater than the intercellular gap of the healthy tissue, while it is smaller than the pores found within the tumor vasculature. Thus, nanoscale architecture may have selectively targeted Magh-Rh_2_Cit and Magh-Cit to the tumor, resulting in higher inhibition of tumor growth compared to Rh_2_Cit [17]. Furthermore, neoplastic cells require higher amounts of micronutrients, such as iron, for cell division, possibly leading maghemite NPs to target the tumor [39]. This could explain the significant differences observed in serum iron of Magh-Rh_2_Cit and Magh-Cit groups in relation to healthy animals and control and Rh_2_Cit (Table 1).

**Table 1:** Effects of Rh_2_Cit, Magh-Rh_2_Cit, and Magh-Cit on hematology and biochemical parameters of female Balb/c mice 19 days after animals’ tumor transplantation

Treatments	Healthy	Control	Rh_2_Cit	Magh-Rh_2_Cit	Magh-Cit	*p* values
Hematology						
RBCs (×10^6^/mm^3^)	9.61 ± 0.05	9.06 ± 0.15*a	7.55 ± 1.34*a	9.43 ± 0.16	9.47 ± 0.12	0.046
HGB (g/dL)	14.88 ± 0.54	14.62 ± 0.10	12.16 ± 2.31	15.12 ± 0.29	14.46 ± 0.19	0.300
HCT (%)	45.30 ± 0.37	43.58 ± 0.55	35.16 ± 6.23*a	44.44 ± 0.73	44.00 ± 0.50	0.084
MCV (fL)	47.15 ± 0.44	48.17 ± 0.86	46.70 ± 0.34	47.12 ± 0.25	46.50 ± 0.55	0.281
MCH (pg)	15.48 ± 0.49	16.13 ± 0.25	15.72 ± 0.70	16.04 ± 0.09	15.28 ± 0.14*b, d	0.094
MCHC (g/mL)	32.88 ± 1.33	33.57 ± 0.27	33.68 ± 1.73	34.04 ± 0.18	32.88 ± 0.27	0.878
Platelets (×10^3^/mm^3^)	503.25 ± 53.63	484.50 ± 23.75	393.80 ± 86.04	428.20 ± 74.85	509.20 ± 43.54	0.592
WBC (/mm^3^)	5193 ± 1688	15,738 ± 4593	18,462 ± 13,158	13,576 ± 2685	14,768 ± 3929	0.249
Lymphocytes (%)	84.25 ± 1.93	8.17 ± 5.27*a	6.00 ± 3.27*a	13.60 ± 10.09*a	4.40 ± 2.68*a	0.035
Neutrophils (%)	12.25 ± 1.65	85.33 ± 4.65*a	90.40 ± 3.31*a	80.40 ± 9.53*a	92.40 ± 2.38*a	0.014
Eosinophils (%)	0.75 ± 0.25	0.33 ± 0.21	0.60 ± 0.25	0.60 ± 0.25	0.20 ± 0.20	0.451
Monocytes (%)	2.75 ± 1.18	6.00 ± 1.81	2.40 ± 0.68	5.20 ± 1.24	2.80 ± 0.37	0.203
Biochemical						
ALT (U/L)	32.40 ± 3.04	24.29 ± 2.88	30.20 ± 3.25	23.00 ± 0.89	31.00 ± 2.71	0.087
Creatinine (mg/dL)	0.40 ± 0.00	0.21 ± 0.01*a	0.22 ± 0.02*a	0.20 ± 0.00*a	0.22 ± 0.02*a	0.001
Serum Fe (μg/dL)	180.80 ± 7.37	130.00 ± 7.21*a	133.80 ± 13.43*a	148.40 ± 12.09	161.33 ± 4.59	0.004

Legend: *RBC* red blood cells, *HGB* hemoglobin, *HCT* hematocrit, *MCV* mean corpuscular volume, *MCH* mean corpuscular hemoglobin, *MCHC* mean corpuscular hemoglobin concentration, *WBC* white blood cells, *ALT* alanine transaminase

*Values are represented as mean ± standard error (*p* < 0.05)

^a^Significant compared to the healthy group

^b^Significant compared to the control group

^c^Significant compared to the group treated with Magh-Rh2Cit

The antineoplastic effect was also showed by the morphometry of the tumors’ necrotic areas, with an increase in its percentage mean in all treatments (Fig. 1c). Even though data presented non-significant values compared to the control, these analyses corroborated tumor volume measurements, except that Magh-Rh_2_Cit promoted a higher percentage of tumor necrosis than Magh-Cit (Fig. 1a, c). This result was possibly due to the presence of rhodium, which induced a better antitumor effect. However, the fact that Rh_2_Cit treatments caused a smaller necrosis area than Magh-Cit might be related to the greater dispersion of free rhodium through the body, as described above (Fig. 1e–h).

Furthermore, the antitumor effect of Magh-Cit may be related to the fact that citric acid is a good antioxidant [40] and the most effective inhibitor of glycolysis, making it useful to fight cancer. For this purpose, citrate can be applied as an inhibitor of the phosphofructokinase enzyme, the pyruvate dehydrogenase complex, and the succinate dehydrogenase enzyme of the Krebs cycle, blocking glycolysis and, thus, resulting in limited metabolism of the mitochondria and low reproduction capacity of the cell in general [41]. Accordingly, citric acid has been described as an antineoplastic treatment in patients with terminal medullary thyroid cancer [42] and terminal peritoneal mesothelioma [43]. In this context, the use of Magh-Rh_2_Cit resulted in the highest mean percentage of the necrosis area and may have caused a greater antitumor effect in mice due to three activities: the local rhodium effect, the NP-mediated drug delivery to the tumor, and the inhibition of glycolysis by citrate.

### Histopathological analyses of the mammary gland and tumors

**Fig. 2:**
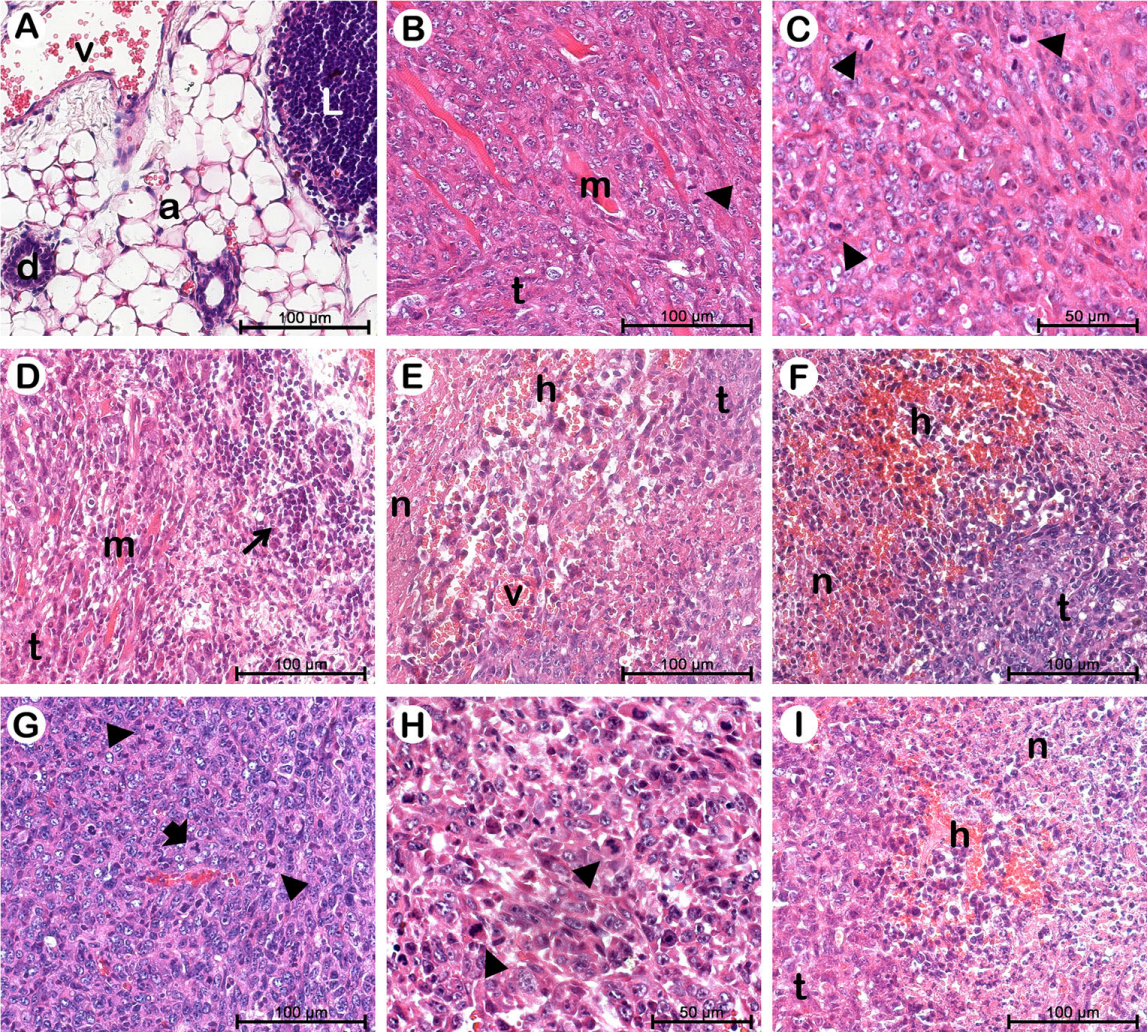
Representative histopathology of mammary gland and tumors developed from Balb/c mice bearing 4T1 breast carcinoma in non-treated and treated groups. Tumor slides were stained with H&E. **a** Healthy group. **b**, **c** Control. **d**, **e** Rh_2_Cit. **f**, **g** Magh-Rh_2_Cit. **h**, **i** Magh-Cit. Adipocyte (*a*). Duct (*d*). Hemorrhage (*h*). Lymph node (*L*). Muscle fibers (*m*). Necrosis areas (*n*). Tumor (*t*). Blood vessel (*v*). Mitosis (*arrow heads*). Infiltration of inflammatory cells (*arrow*). Three spindle poles (*large arrow*)

Histological analysis of the mammary gland of healthy mice showed peculiar parenchyma and stroma with ducts surrounded by connective and adipose tissue, and the presence of a lymph node (Fig. 2a). In the 4T1-bearing mice, the neoplastic tissues in all groups presented malignant anaplastic tumors with cells not specialized and distinct from the original parenchyma (Fig. 2b–i). The fast growth of cancer cells upon the parenchyma and stroma of breast tissue is related to morphological changes, such as loss of polarity due to pleomorphic cells and a large amount of typical and atypical mitosis, with three spindle poles, as shown in Fig. 2g. These features are related to the high malignancy of 4T1 cells, which were described as an invasive lineage, representing a suitable model to evaluate the efficacy of anticancer drugs due to its similarities with metastatic human breast cancer [29].

Tumors were more invasive in the control group (water administration). This was evidenced by the presence of more local invasion in the muscle fibers beneath the tumors (Fig. 2b). A dense inflammatory reaction was seen around tumors in the Rh_2_Cit group compared to other groups, corroborating the findings of our previous study [24]. Moreover, various tumor cells were observed in a process of necrosis with hemorrhagic areas in tissues of Rh_2_Cit, Magh-Rh_2_Cit, and Magh-Cit groups (Fig. 2e, f, i). Necrotic tissues showed disorganized cellular debris with indistinct boundaries, pyknotic nuclei, and eosinophilic cytoplasm, forming a homogeneous amorphous tissue (Fig. 1d).

### Systemic toxicity assessment in mice bearing 4T1 breast carcinoma

**Fig. 3:**
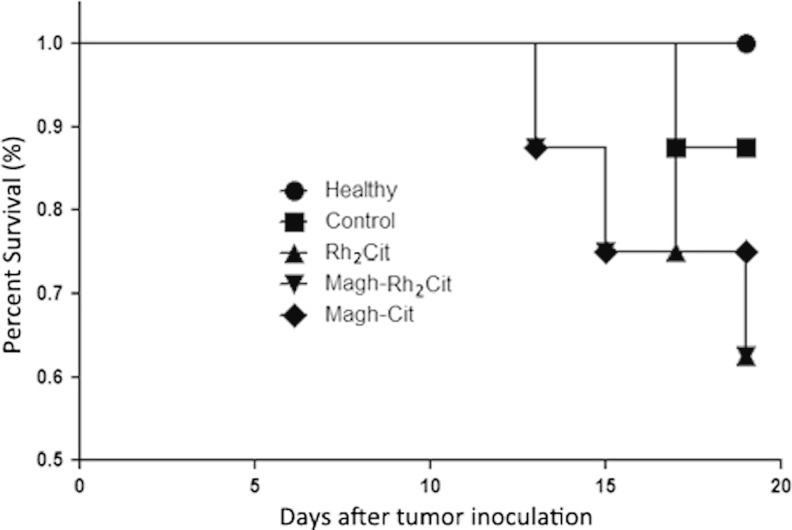
Survival curve for tumor-bearing mice during 19 days of non-treated and treated groups. Animals received intravenous administration in tail vein of three doses of 0.5 mg/kg (1.5 mg/kg total dose) rhodium (II) citrate for Rh_2_Cit and Magh-Rh_2_Cit treatments, and the same iron concentration and nanoparticle amount for Magh-Rh_2_Cit and Magh-Cit. Values represent mean values ± standard error (*n* = 8/each group). Animals without tumor and without treatment (healthy) were also included in this study as negative control group

During the whole experimental period (19 days), the in vivo survival rates of all animals were examined. Deaths started on experimental day 13 after tumor implantation and occurred until euthanasia, totalizing nine deaths in all groups with tumor. The final survival rates of mice were of 100 % in the healthy group, 87.5 % (one death) in the tumor control group, 62.5 % (three deaths) in the Rh_2_Cit and Magh-Rh_2_Cit groups, and 75 % (two deaths) in the Magh-Cit group (Fig. 3). However, no evident alterations in clinical parameters were found during the whole experimental period, which indicated the good tolerance of mice to treatments. Thus, the survival rates in mice treated with these formulations should be attributed to the natural invasiveness of the tumor and not to the treatments themselves.

Values of erythrogram of mice in the control group and those treated with Rh_2_Cit, Magh-Rh_2_Cit, and Magh-Cit remained similar to those of healthy mice. Still, a significant reduction in RBC was observed in the control and Rh_2_Cit groups and in HCT in the Rh_2_Cit group (Table 1). However, although concurrent controls are used as the primary comparison for interpretation of treatment-related changes, comparison to reference intervals may also be useful to put hematological changes in perspective [44]. In this context, we concluded that there were no hematotoxic effects for all experimental groups, since RBC, HCT, and the other variables of the hemogram remained inside the reference range described for female mice (6.5–10.1 × 10^6^/μL for RBC and 32.8–48 % for HCT) [45]. The hematological changes could be related to the development of the tumor, according to these reference values. This observation is supported by the leukogram data, whereas there were no significant differences regarding the WBC, compared to healthy animals.

Generally, in all groups, we observed a significant decrease in the percentage of lymphocytes with a concomitant increase in the percentage of neutrophils after tumor implantation (Table 1), corroborating the results of other studies in our group [8, 37, 49]. The relative WBC counts have been reported as a non-relevant parameter, as it is the absolute counts that must be reported and interpreted [46]. Overall, the results support the finding that there were no hematotoxic effects in all treatment groups.

**Fig. 4:**
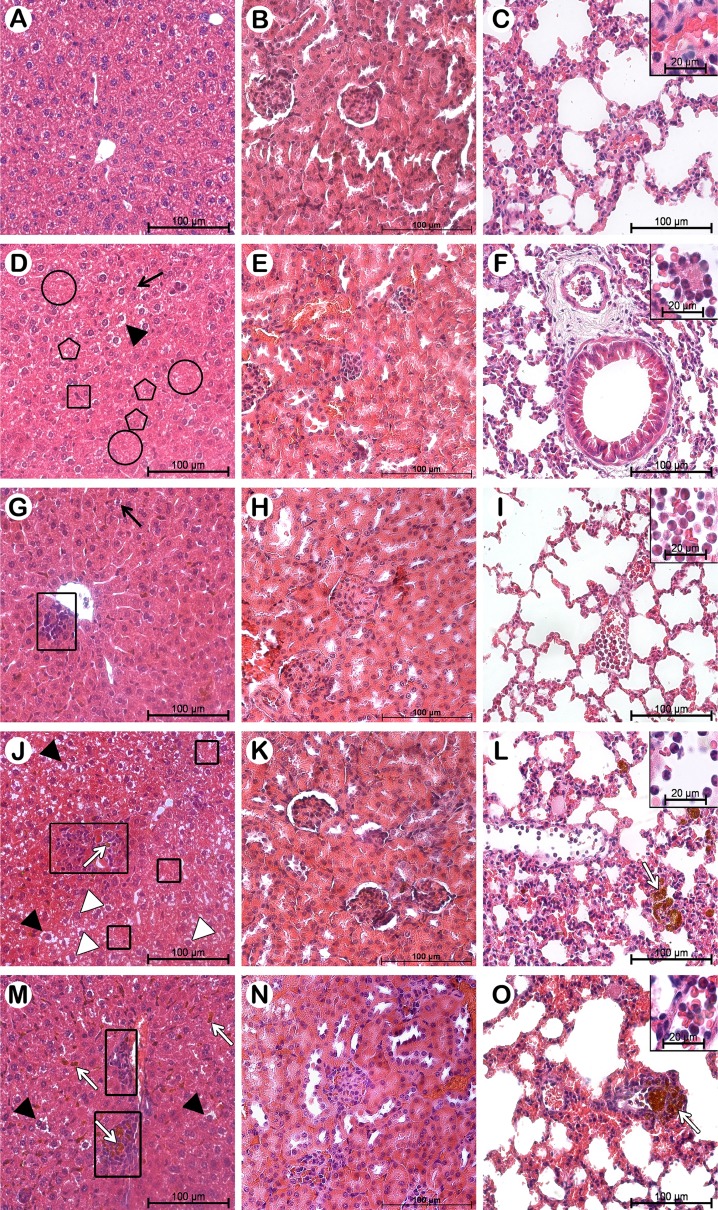
Representative histopathology of liver, kidney, and lung from healthy Balb/c mice and Balb/c mice bearing 4T1 breast carcinoma in non-treated and treated groups. The animals were treated with the same Rh_2_Cit concentration (1.5 mg/kg total dose), and they were euthanized on the 19th day after tumor inoculation. In general, no morphological alterations were found in the kidneys and lungs of mice. Increased neutrophilis were found in all mice bearing mammary carcinoma, and we also observed nanoparticles in lung tissue of mice treated with Magh-Rh_2_Cit and Magh-Cit. Organ sections were stained with H&E. Healthy mice (**a**–**c**) and mice bearing 4T1 breast carcinoma without treatment (**d**–**f**) or treated with Rh_2_Cit (**g**–**i**), Magh-Rh_2_Cit (**j**–**l**), and Magh-Cit (**m**–**o**). Fibrosis (*circle*). Hepatocyte degeneration (*square*). Inflammation (*rectangle*). Nanoparticles (*white arrow*). Nucleus degeneration (*pentagon*). Pyknotic nuclei (*arrow*). Vacuolization (*arrow head*). Nucleus hypertrophy (*white arrow head*)

Our histopathological analyses of the lung corroborated the relative WBC counts, showing alveolar wall vessels with an increase in the number of neutrophils in animals bearing mammary carcinoma (Fig. 4f, i, l, o), while the healthy group presented a low concentration of leucocytes (Fig. 4c). However, the rise in WBC was restricted to vessels, with no evident perivascular infiltration of inflammatory cells, even in animals treated with Magh-Rh_2_Cit and Magh-Cit, which presented NPs inside blood vessels and in perivascular parenchyma (Fig. 4l, o). Moreover, no foamy cells, pulmonary fibrosis, or presence of granulomatous lesions were observed in lung tissues of all treatment groups (Fig. 4). Thus, we concluded that our nanoformulations were non-toxic, since there was no morphological change in the lung tissue in all experimental groups.

Along with the hemogram, evaluation of the serum biochemical parameters is crucial for identifying the possible occurrence of toxicity induced by a new drug, and it must be correlated with histopathology tests [46]. Thus, serum levels of ALT were measured to assess possible dysfunctions in the hepatobiliary system [46, 47] arising from the tumor implantation and/or treatments. Serum creatinine was used as a kidney function test [46], and the results were compared to the histopathological analyses of these organs (Fig. 4). ALT is a cytoplasmic enzyme in which the serum activity is increased due to leakage across damaged cytoplasmic membranes. In mice, ALT is found in the highest concentration in the liver, although its activity has also been demonstrated in the intestine, kidney, heart, muscle, and brain. Despite its widespread tissue distribution, ALT is widely considered to be a serum biomarker of hepatocellular injury [46], being used as an indicator of drug-induced liver hepatotoxicity [48]. In this study, ALT levels showed no significant statistical differences (Table 1), as all groups were within the reference values reported for female Balb/c mice [46].

**Fig. 5:**
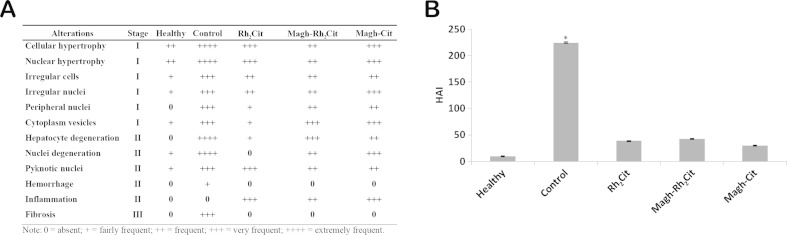
Histopathological alterations found in the liver of Balb/c mice bearing 4T1 breast carcinoma in non-treated and treated groups. The animals were treated with the same Rh_2_Cit concentration (1.5 mg/kg total dose), and they were euthanized on the 19th day after tumor inoculation. Liver sections were stained with H&E. **a** Alterations were classified as progressive stages (I, II, and III) for the deterioration of liver functions. **b** Histopathological alteration index (HAI) was divided into 0–10 = normal tissue, 11–20 = mild to moderate damage to the tissue, 21–50 = moderate to severe damage to the tissue, 51–100 = severe damage to the tissue, and greater than 100 = irreparable damage to the tissue. *Asterisk* indicates significant differences (*p < 0.05)

On the other hand, compared to healthy animals’ liver, we verified a significantly increased HAI in the control group and a higher value in the treatment groups (Fig. 4a, d, g, j, m). It was observed that the untreated animals (control group) had HAI above 100, indicating that the liver function was irreparably affected by signs of fibrosis. These changes in the liver, classified as stage III, prevent the restoration of its functional structure (Fig. 5b) [34]. For the animals treated with Rh_2_Cit, Magh-Rh_2_Cit, and Magh-Cit, the mean of liver tissue changes was between 21 and 50, indicating the presence of liver abnormalities ranging from moderately to heavily damaged [49] (Fig. 5a).

In the measurements of the hepatocytes, the control group had significantly increased nucleus area and volume compared to the healthy group, causing liver hypertrophy, but this was improved by the treatments. Animals treated with Rh_2_Cit and Magh-Cit showed a significant reduction in the hepatocyte diameter (Table 2). These results could be associated with the presence of inflammatory infiltrate observed near blood vessels, mainly close to the NP agglomerations (Fig. 4j, m). These inflammatory infiltrates, known as inflammatory pseudotumors (IPTs), are the expression of diverse inflammatory processes that may be accompanied by a tumor-like mass [50]. The IPTs in the hepatic tissue may suggest that NPs activate the phagocytic activity to help in removing the accumulated NPs. Thus, this process could represent a defense mechanism of detoxification which may be correlated with the amount of injury to the hepatic tissue [51]. In the untreated animals (control group), the tumor growth induced irreversible pathological changes, with no reestablishment of the functional structure of the liver due to the presence of signs of fibrosis (Fig. 4d). The histopathological changes in the liver may therefore have been caused by breast tumor growth, leading to physiological disorders.

**Table 2:** Quantitative parameters of hepatocyte measurements in healthy Balb/c mice and mice bearing 4T1 breast carcinoma without treatment or treated with Rh_2_Cit, Magh-Rh_2_Cit, and Magh-Cit

Groups	Healthy	Control	Rh_2_Cit	Magh-Rh_2_Cit	Magh-Cit
Hepatocyte area (μm^2^)	84.7 ± 21.1	85.7 ± 7.8	72.3 ± 12.9	83.5 ± 10.4	75.3 ± 12.7
Hepatocyte volume (μm^3^)	600.3 ± 234.1	599.2 ± 85.8	468.9 ± 129.0	577.5 ± 116.5	497.1 ± 131.0
Cytoplasm area (μm^2^)	75.6 ± 20.3	72.0 ± 7.6	62.5 ± 11.9	73.4 ± 9.3	66.1 ± 12.0
Cytoplasm volume (μm^3^)	508.2 ± 201.4	462.0 ± 73.0	376.9 ± 105.0	476.3 ± 91.6	409.9 ± 109.5
Cytoplasmic/nuclear ratio (μm^2^)	8.3 ± 1.6	5.2 ± 1.7	6.32 ± 2	7.2 ± 1.8	7.2 ± 1.1
Nucleus area (μm^2^)	9.0 ± 1.6	13.7 ± 1.7*a	9.8 ± 2.0*b	10.0 ± 1.8*b	9.1 ± 1.1*b
Diameter of nucleus (μm)	3.7 ± 0.2	4.1 ± 0.4	4.0 ± 0.3	4.0 ± 0.3	3.9 ± 0.3
Nucleus volume (μm^3^)	20.8 ± 5.3	38.3 ± 7.3*a	23.7 ± 7.6*b	24.3 ± 6.7*b	20.9 ± 3.8*b

Letters indicate significant differences in 2-by-2 comparisons detected by the Tukey test, with a = significant compared to the healthy group and b = significant compared to the control group. Results are expressed as mean ± SE of the mean

*p < 0.05

Creatinine is a degradation product of creatine and creatine phosphate and represents an end product of muscle metabolism. Pathologically, elevated serum creatinine levels can be caused by prerenal, renal, and postrenal conditions. Prerenal causes include increased protein catabolism, such as that which occurs in inflammation or with high-protein diets. Renal causes are usually associated with conditions that compromise 70–75 % of functional renal mass, and postrenal causes include any cause that results in obstruction of the lower urinary system [46]. In the present study, the creatinine levels of all groups of mice bearing breast cancer was significantly lower compared to healthy mice (Table 1). Thus, we found no impairment of kidney function by the treatments, and the change in creatinine levels could be related to the development of the tumor since a decrease in creatinine levels has been reported as having no clinical significance [52]. Our histopathological analyses of the kidneys are supported by this conclusion, since no morphological alterations in the kidneys were found (Fig. 4b, e, h, k, n). These data are in agreement with our previous report with the same formulations when intratumorally administered [24].

## Conclusion

In summary, we demonstrated by intravenous administration that Magh-Rh_2_Cit and Magh-Cit promoted a significant decrease in tumor area and did not induce toxicity effects in tested experimental conditions. No studies about the systemic action of rhodium (II) citrate-loaded maghemite nanoparticles have been carried out, making this report a suitable starting point for exploring the therapeutic potential of these compounds in treating breast cancer.

## Abbreviations

SPIOs: Superparamagnetic nanoparticles
NPs: Nanoparticles
RES: Reticuloendothelial system
EPR: Enhanced permeability and retention
Rh_2_Cit: Free rhodium (II) citrate
Magh-Rh_2_(H_2_cit)_4_ or Magh-Rh_2_Cit: Rhodium (II) citrate-loaded maghemite nanoparticles
Magh-Cit: Maghemite nanoparticles coated with citrate
DMEM: Dulbecco’s modified Eagle’s medium
FBS: Fetal bovine serum
i.v.: Intravenous
ALT: Alanine aminotransferase
AVMA: American Veterinary Medical Association
PBS: Phosphate-buffered saline
HE: Hematoxylin-eosin
HAI: Histopathological alteration index
SEM: Standard error of mean
CNPq: Brazilian National Council for Technological and Scientific Development
FAPDF: Foundation to Support Research in the Federal District
CAPES: Coordination for Further Training of Graduate Staff
UnB: University of Brasília
